# Characteristics of Periodontal Tissues in Prosthetic Treatment with Fixed Dental Prostheses

**DOI:** 10.3390/molecules26051331

**Published:** 2021-03-02

**Authors:** Anna Avetisyan, Marina Markaryan, Dinesh Rokaya, Marcos Roberto Tovani-Palone, Muhammad Sohail Zafar, Zohaib Khurshid, Anna Vardanyan, Artak Heboyan

**Affiliations:** 1 Department of Therapeutic Stomatology, Faculty of Stomatology, Yerevan State Medical University, Street Koryun 2, Yerevan 0025, Armenia; avetisyan_an@mail.ru (A.A.); marmiga@mail.ru (M.M.); 2 Department of Clinical Dentistry, Walailak University International College of Dentistry, Walailak University, Bangkok 10400, Thailand; 3 Department of Pathology and Legal Medicine, Ribeirão Preto Medical School, University of São Paulo, Ribeirão Preto 14049-900, Brazil; 4 Department of Restorative Dentistry, College of Dentistry, Taibah University, Al Madinah, Al Munawwarah 41311, Saudi Arabia; 5 Department of Dental Materials, Islamic International Dental College, Riphah International University, Islamabad 44000, Pakistan; 6 Department of Prosthodontics and Implantology, College of Dentistry, King Faisal University, Al-Hofuf, Al-Ahsa 31982, Saudi Arabia; drzohaibkhurshid@gmail.com; 7 Department of Prosthodontics, Faculty of Stomatology, Yerevan State Medical University, Street Koryun 2, Yerevan 0025, Armenia; annavardanyan@yahoo.co.uk

**Keywords:** metal ceramic, zirconium, CAD/CAM, dental prosthesis, plaque index, gingival biotype, periodontal index, oral hygiene, gingival pocket

## Abstract

The objective of the present study was to investigate the effects of various types of fixed prostheses on periodontal tissues and explore the association of gingival biotype and gum recession in relation to prosthesis types. The study participants (N = 95) were divided into three groups based on the type of dental prosthesis: Group-I: cobalt-chrome (Co-Cr) ceramic prosthesis fabricated by the conventional method (n = 35); Group-II: consisted of patients with Co-Cr ceramic prostheses fabricated by a computer-aided design and computer aided manufacturing (CAD/CAM) technique (n = 30); and Group-III: zirconia-based prostheses fabricated by the CAD/CAM technique (n = 30). Following the use of prostheses, periodontal examinations were performed using the Community Periodontal Index (CPI) and Modified Approximal Plaque Index (MAPI). In addition, the gingival biotype was examined using a probe transparency method. The Statistical Package for the Social Sciences (SPSS), Version 20 (IBM Company, Chicago, IL, USA), was used to analyze the results, and the significance level was set at *p* = 0.05. It showed the MAPI results after the use of prosthetic rehabilitation for 12 months of periodontitis in 87.9% ± 15.4 of patients in Group-I, in 80.6% ± 17.97 in those in Group-II, and in 62.5% ± 21.4 in those in Group-III (*p* < 0.01). The CPI index results indicated a high prevalence of periodontal disease in all groups. The number of people with healthy periodontium constituted 17.1% of patients in Group-I, 24.2% in Group-II, and 37.1% in Group-III. Our study concluded that prosthetic treatment with periodontal diseases showed better outcomes while using dental prostheses fabricated by the CAD/CAM technique compared to the conventionally fabricated dental prostheses. The thin gingival biotype is more often associated with gingival recession than the thick biotype.

## 1. Introduction

The functional disorders of the stomatognathic system due to periodontal diseases are five times more frequent than those resulting from dental caries [1]. Periodontal conditions result in loss of teeth requiring dental prosthesis. The unrestored edentulous space and alveolar ridges may lead to several significant changes, such as the impairment of biomechanics in the dentofacial system, poor aesthetic, deterioration of periodontal tissues, and negative effects on the general health and social behaviors of the patient [2]. There are various methods of restoration of partially edentulous patients, and the fixed dental prostheses fabricated using metal alloys and porcelain are one of the common methods for the restoration of missing teeth [3,4].

The goal of the fixed prostheses is to control oral disease while restoring aesthetics and function with durable, biocompatible restorations. Teeth proportions, crown weight/length ratio, gingival zenith, etc., play an important role in dental aesthetics [5,6]. In addition, knowledge of the responses of periodontal tissues to fixed prostheses and periodontal aesthetics are important in the development of treatment plans with predictable prognoses and patients’ acceptance [7]. Biofunctionality and the harmony between the prosthesis and the periodontium is important for the aesthetics and longevity of the prosthesis [7]. In this regard, several factors, such as the prosthesis design, pontic design, occlusion, and biomaterial may contribute and should be considered while planning the fixed prosthodontic treatment [8].

Furthermore, the preparation of the margin, contour, and emergence profile of the prosthesis can influence the gingival tissues’ response to the prosthesis. However, improperly fabricated prosthesis may either damage the health oral tissues or exacerbate existing periodontal conditions such as gingivitis, periodontitis, and occlusal trauma. Restorative materials can affect biofilm formation, since rough and irregular surfaces create a favorable environment for bacterial colonization [9,10,11]. Similarly, ignoring the phenotype of the gingival tissues while planning a fixed prosthesis may exaggerate the existing lesions [12,13]. For example, the depth of gingival sulcus, the thickness of gingival epithelium (gingival biotype), as well as the location of the alveolar ridge varies from one patient to the other and should be considered during the treatment planning [14,15,16]. 

The accurate marginal and internal fit are essentially required for the success and longevity of fixed dental protheses. An improper crown margin facilitates plaque accumulation, gingival sulcular fluid flow, and bone loss, which may lead to microleakage, recurrent caries, periodontal diseases, and ultimately the failure of prosthetic restorations [17]. Therefore, the protection of the marginal gingiva, tissue-biomaterials interaction, and clinical assessment of the oral health, particularly of the periodontal tissues, are important for the longevity and clinical success of the fixed partial dentures fabricated using different biomaterials.

Thus, the objective of the present study was to investigate the effects of various types of fixed partial prostheses fabricated using different techniques on the periodontal tissues. In addition, the association of gingival biotype and gum recession in relation to prosthesis types was explored.

## 2. Materials and Methods

### 2.1. Participants and Study Groups 

The present clinical study included a total of 95 patients with a mean age of 29 (18–40 years old) attending the “Nord KS” dental clinic in Yerevan, Republic of Armenia, from June 2019 to July 2020 and requiring prosthodontic treatment. All the participants were divided in to three study groups (Table 1).

The inclusion criteria included:

- Age of the participants 18–40 years,
- Both males and females,
- Healthy without any complaints or periodontal pathology,
- Subjects with gingivitis,
- Subjects with periodontitis,
- Patients requiring fixed prostheses.

The exclusion criteria included:

- Patients with systemic diseases,
- Complete missing teeth,
- Pregnant women,
- Smokers.

The prostheses types used in this study were cobalt-chrome ceramic prostheses fabricated by the conventional method, cobalt-chrome ceramic prostheses fabricated by a computer-aided design and computer aided manufacturing (CAD/CAM) technique, and zirconia-based prostheses fabricated by the CAD/CAM technique. The restorations of all groups were fixed with the same glass ionomer luting material (GC Fuji I® Glass Ionomer Luting Cement, GC America Inc) with an acceptable fixation protocol [18]. 

Ethical clearance was obtained from the Ethics Committee of Yerevan State Medical University, Republic of Armenia (IRB APPROVALN12-5/2019, 13 June 2019). The research protocol and the clinical procedures were thoroughly explained, and informed consent was obtained from the individuals.

### 2.2. Evaluation of Periodontal Health

The dental examiners who participated in patients’ recruitment received 3-day training and calibration sessions. Considering the high prevalence of inflammatory periodontal diseases, the state of periodontal tissues, and the gingival biotypes, trained professionals assessed patients before and a year after prosthetic rehabilitation using the verified assessment tools, including the Community Periodontal Index (CPI index) and the probe transparency method (TRAN), respectively [19]. Briefly, the CPI was recorded for each sextant using a 0.5 mm ball-ended probe with color markers at 3.5 and 5.5 mm. According to the World Health Organization (WHO) recommendations, the pressure applied in the probe did not exceed 20 g [19]. Ten index teeth (17, 16, 11, 26, and 27 in the maxilla, and 47, 46, 31, 36, and 37 in the mandible) in six sextants (17–14, 13–23, 24–27, 37–34, 33–43, and 44–47) were evaluated, and scores were ascribed to each sextant on the following basis:

- score 0 — no signs of disease
- score 1 —gingival bleeding after gentle probing
- score 2 — presence of supra or subgingival calculus or other plaque retentive factors
- score 3 — 4 to 5 mm deep periodontal pockets
- score 4 — 6 mm or deeper periodontal pockets

Molars were examined in pairs and the highest score was recorded for each sextant. Sextants were examined for the presence of at least two teeth, and a total of six sites were examined per tooth: mesial, midline, and distal on both vestibular and lingual/palatal surfaces. We excluded the sextants with no teeth or with teeth that could not be examined. The screening examination also included additional indices as secondary outcome measures—the Modified Approximal Plaque Index (MAPI) as shown in Table 2 [20]. MAPI was used to analyze the distribution of the visible plaque on the surfaces of all incisors, canines, premolars and first molars. 

MAPI values were calculated as follows: 

points/number of teeth per patient × 100%.

Probing depth is defined as the distance from the free gingival margin to the bottom of the pocket/sulcus [21]. Measurements regarding gingival biotype were made until two duplicate values were registered. The gingival biotype was considered thin in cases where the measurement was < 1.0 mm and thick if it was > 1.0 mm. Based on the TRAN method, the gingival biotype was considered thin if the outline of the probe could be shown through the gingival margin from the sulcus. Measurements were done on the mid-vestibular aspect of each tooth [22].

No periodontal therapy was done during 12 months of observation period. Only professional oral hygiene measurements were done in all groups before and 6 months after prosthetic treatment. 

### 2.3. Statistical Analysis

The data were analyzed using the statistical software SPSS 20 (IBM Company, Chicago, IL, USA), and the statistical analysis was performed using a paired sample t-test from a *p* value of 0.05 was considered as statistically significant. Percentages were used as relative indicators for some categories of variables.

## 3. Results

In the present study, a total of 24 subjects were healthy without any complaints or periodontal pathology, while 39 patients were diagnosed with gingivitis and 32 patients with periodontitis. Gingivitis and periodontitis were seen in subjects at 6 months following the insertion of fixed dental prostheses. The subjects were followed up 6 months and 12 months after the treatment.

The results of the MAPI data in all treatment groups before and after prosthetic treatment are shown in Table 3. The data obtained before the prosthetic rehabilitation indicated unsatisfactory oral hygiene, both in gingivitis and chronic periodontitis patients in the Group-I. The indicators in Group-II also revealed unsatisfactory oral hygiene in patients with signs of gingivitis and periodontitis, the mean value of MAPI was 76.4% ± 28.8 and 96.5% ± 8.6 (*p* < 0.01), respectively, and the patients without periodontal pathology in this group were only 24.2%. In Group-III, we observed that patients with chronic gingivitis had a mean value of MAPI of 84.9% ± 32.6 before the prosthetic rehabilitation. In patients with periodontitis, the mean value was 93.3% ± 13.9 (*p* < 0.003), which also corresponds to unsatisfactory oral hygiene. Oral hygiene conditions were clinically manifested by a hyperemia of gingival papilla with transition to the gingival margin.

Regarding the period of 1 year after the rehabilitation, we found a change in the mean value of MAPI in the Group-I patients with gingivitis, which was 76.4% ± 17.4 (*p* < 0.01). This value is interpreted as “poor oral hygiene” and corresponds to a statistically significant difference in comparison with the indicators of this group before the rehabilitation (*p* < 0.01). In addition, we also observed a change in the index of oral hygiene in patients with chronic periodontitis, whose mean value was 87.9% ± 15.4 (*p* < 0.01), and it was classified as “poor hygiene”. The difference in the indicators of this group before and after the rehabilitation was reliably confirmed (*p* < 0.01). In the Group-II patients, the hygiene index in those with gingivitis decreased to 44.8% ± 26.7 after the rehabilitation (*p* < 0.01), and their oral hygiene was classified as “moderately poor”. In the patients with periodontitis the index decreased to 80.6% ± 17.97 (*p* < 0.01), which corresponds to a “poor oral hygiene”. The decrease in the indicators was statistically significant (*p* < 0.01). The data obtained 12 months after the prosthetic rehabilitation in patients of Group-III with gingivitis revealed a significant decrease in the mean value of MAPI to 40.7% ± 21.4, which was interpreted as “moderately poor oral hygiene”. However, despite a significant decrease in the indicator to 62.5% ± 21.4, the level of hygiene remained poor (*p* < 0.01) in patients with periodontitis. Furthermore, in patients with periodontitis, when comparing results in the study groups one year after the prosthetic rehabilitation, the best indicators were recorded in groups of both patients with Co-Cr ceramic and zirconia-based prostheses fabricated by the CAD/CAM technique (t = 5.3 and t = 2.7, respectively *p* < 0.01).

In the present study, the mean values of MAPI in patients with a healthy periodontium were distributed into three groups. In Group-I the indicators improved by 5%, but within the same level, i.e., poor oral hygiene before the prosthetic rehabilitation was 75.17% ± 7.9 and after the prosthetic rehabilitation was 70.3% ± 8.4 (*p* < 0.05). In the observation Group-II and Group-III, hygiene indicators decreased from poor to moderately poor oral hygiene, from 71.95% ± 38.2 and 71.8% ± 39.8 before the prosthetic rehabilitation to 51.8% ± 30.9 and 41.3% ± 28.2 one year after the prosthetic rehabilitation (*p* < 0.01 and *p* < 0.001, respectively). The assessment of the periodontal tissue status using the CPI index revealed a high prevalence of periodontal diseases in all observed groups. This is due to a small number of patients with healthy periodontal status, which was observed in 17.1% of the cases in Group-I patients and, in 24.2% and 37.1%, in Group-II and Group-III patients, respectively. While studying the intensity of periodontal disease, we found that the CPI index indicated a high incidence of periodontal disease in patients of the Group-I before the prosthetic rehabilitation.

**Figure 1:**
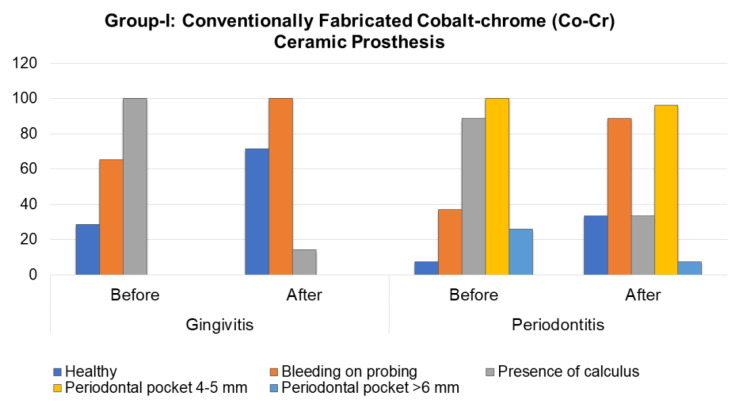
Periodontal status before and after the prosthetic rehabilitation in Group-I patients.

Dental plaque associated with bleeding of the gums (with an intensity of 4.14 ± 1.57, present in 1.43 ± 1.6 segments per patient (*p* < 0.05)) was the most common sign in the first group in patients with gingivitis before the prosthetic rehabilitation. After the rehabilitation, a significant redistribution of the index structure was observed. A decrease of 14.3% in the mean percentage of sextants with dental plaque was verified, while the mean percentage of healthy sextants increased to 71.5%. However, an increase in the mean percentage of sextants with bleeding to 100%, with an intensity of 4.57 ± 0.8 (*p* < 0.01), was observed (Figure 1).

Inflammatory processes were found in this group in patients with periodontitis with bleeding while brushing, bad breath, hypersensitivity to temperature changes, and presence of hard dental plaque that could not be removed by brushing. The clinical examination revealed signs of congestive hyperemia of the gingival papillae and exposure of tooth necks up to one fourth of the root length. Periodontal pockets were found with an average number of segments of 2.3 ± 1.07 (*p* < 0.01) per patient examined with this symptom. With age, the percentage of people with clinical gingival pockets of 6 mm or more increased, as well as the average number of affected segments 0.37 ± 0.69 (*p* < 0.01) per patient examined. After the prosthetic rehabilitation, a decrease in the signs of periodontal lesions was observed. The mean percentage of sextants with plaque was 33.3%, with an intensity of 0.44 ± 0.75 (*p* < 0.01); sextants with a pocket of 4-5 mm amounted to 96.3%, with an intensity of 1.52 ± 0.75 (*p* < 0.01); and sextants with a pocket of 6 mm or more amounted to 7.4%, with an average intensity of 0.11 ± 0.4. This resulted in a significant increase in the number of healthy and bleeding sextants, with a mean percentage of 33.3% with an intensity of 0.59 ±1.1 and 88.9% with an intensity of 2.33 ± 1.4 (*p* < 0.01), respectively. 

The initial CPI assessment in Group-II in patients with gingivitis revealed the signs of periodontal tissue lesions. Bleeding gums during probing were diagnosed on average in 1.0 ± 1.3 sextants and dental plaque was observed in 2.78 ± 2.4 sextants, while 1.78 ± 1.78 sextants remained healthy (*p* < 0.01). The CPI assessment in patients of Group-II with periodontitis revealed a prevalence of sextants with supragingival and subgingival dental plaque (1.69 ± 1.3 sextants; with 4–5 mm pocket - 1.81 ± 0.75 sextants; and excluded sextants - 1.25 ± 1.18 sextants (*p* < 0.001). A pocket of 6 mm or more was observed in 0.25 ± 0.6 sextants (*p* < 0.1), while sextants with bleeding gums corresponded to 0.94 ± 0.9 sextants (*p* < 0.01).

A year after the prosthetic rehabilitation, the mean percentage of healthy sextants in this group in patients with gingivitis increased by 11.1% (with an average intensity of 2.56 ± 2.2; *p* < 0.01). The mean percentage of sextants with dental plaque decreased by 3.5 times in the percentage ratio (*p* = 0.007). The mean percentage of sextants with bleeding gums increased to 100% (*p* < 0.1), with an average intensity of 2.36 ± 1.7. A similar trend was observed in patients of Group-II with periodontitis, who showed a significant decrease in the number of sextants with dental plaque and in pocket depth, from 4–5 mm to 0.69 ± 0.6 and 1.13 ± 0.8, respectively, and an increase in the number of healthy sextants and sextants with bleeding gums to 0.81 ± 0.8 and 2.06 ± 0.6, respectively. This, in turn, indicates a significant improvement in the conditions of periodontal tissues (*p* < 0.01). The percentage ratio of the dynamics of the index is shown in Figure 1.

**Figure 2:**
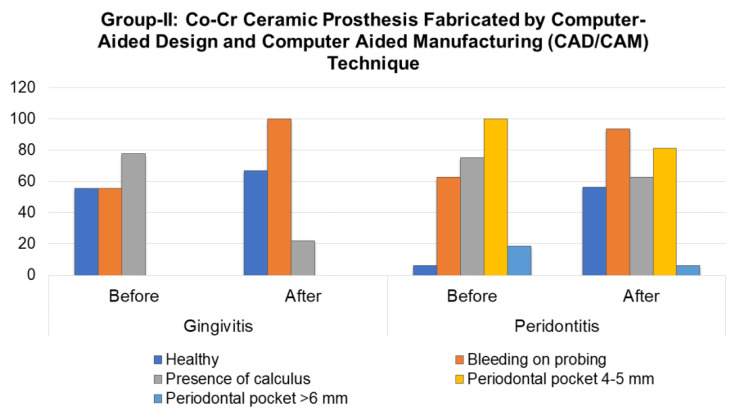
Periodontal status evaluation before and after the prosthetic rehabilitation in observation Group-II patients.

Figure 2 shows the periodontal status evaluation before and after the prosthetic rehabilitation in observation Group-II patients. It shows a pattern similar to that of Figure 1.

**Figure 3:**
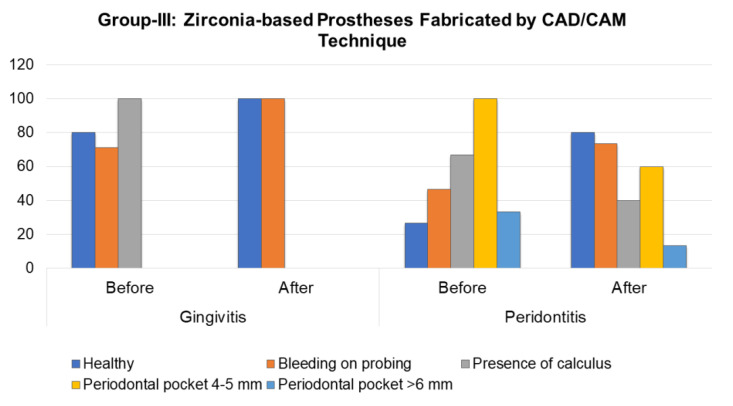
Periodontal status evaluation before and after the prosthetic rehabilitation in observation Group-III patients.

In patients with gingivitis in Group-III, the mean of healthy sextants prior to the prosthetic rehabilitation was 2.0 ± 1.2 (*p* < 0.005), while in sextants with bleeding gums it was 2.0 ± 1.6 (*p* < 0.01). Dental plaque was observed in 1.71 ± 0.8 sextants (*p* < 0.01). One year after the prosthetic rehabilitation, these patients did not show sextants with dental plaque, and the prevalence of bleeding increased to 100%; however, its intensity decreased to 1.43 ± 0.8 (*p* < 0.01). Moreover, the number of healthy sextants was 4.14 ± 1.1 (*p* < 0.003). Figure 3 shows the periodontal status evaluation before and after the prosthetic rehabilitation in observation Group-III patients. The patients with periodontitis prior to the prosthetic rehabilitation had a predominance of sextants with 4–5 mm with periodontal pocket in 2.0 ± 0.8 sextants and presence of dental plaque in 1.2 ± 0.8 sextants (*p* < 0.01). After the prosthetic treatment, a quantitative and percent reliable (*p* < 0.01) increase in healthy sextants or with a sign of bleeding was 80% and 73.4% with an average intensity of 1.67 ± 1.4 and 1.6 ± 1.4, respectively (*p* < 0.001). No significant differences were observed in the CPI scores before and after the prosthetic treatment in patients with healthy periodontium.

The thin periodontal biotype was most often found among the patients of all study groups (Table 4). Furthermore, we verified that the thin gingival biotype was more often associated with gum recession than the thick biotype, which is more resistant to recession and better to cover restoration areas.

After the prosthetic treatment, a change in the gingival biotype characterized by thickening was observed in three patients with periodontitis (18.6%) and one patient with gingivitis (14.3%) in observation Group-I.

## 4. Discussion

The present clinical study investigated the effects of fixed partial prostheses fabricated using various biomaterials and techniques on health and pathological periodontal tissues. The missing teeth in partially edentulous patients were restored using either conventionally fabricated Co-Cr, CAD/CAM-fabricated Co-Cr, or CAD/CAM-fabricated zirconia-based fixed partial dentures. The status of oral health including the periodontal tissues was assessed before and after the insertion of prostheses using various standardized tools, such as CPI and MAPI. In addition, the probe transparency method was used to determine the gingival biotype. The present study demonstrated that the initial level of oral hygiene was poor among the participants of all groups. At the one-year follow up visit after the prosthetic rehabilitation, the average value of MAPI remained virtually unchanged in Group-I and Group-II patients with periodontitis (*p* > 0.5) and Group-I patients with gingivitis. In addition, the comparative analysis of the MAPI data revealed a statistically confirmed improvement in the oral hygiene indicators among the patients with gingivitis in all observation groups. However, the best indices were observed in Group-III patients with gingivitis and periodontitis using the CAD/CAM zirconia-based prostheses (*p* < 0.05 and *p* < 0.001, respectively).

A study done by Manasuri and Shrestha [23] at a tertiary dental care center in Nepal found that there was no association between the wearing of fixed and removable dental prosthesis and the periodontal disease and suggested the need for population-based oral health education programs and plaque control programs to reduce the incidence of periodontal disease. Our study, on the other hand, showed that the type of fixed prosthesis is associated with gingival and periodontal health. Our results are supported by various other studies [24,25,26].

Ercoli and Caton [24] mentioned that prostheses are associated with plaque retention and loss of attachment. Prostheses restoration margins placed within the junctional epithelium and supracrestal connective tissue attachment can be associated with inflammation and, potentially, recession. Adequate periodontal assessment and treatment, appropriate instructions, and motivation in self-performed plaque control and compliance to maintenance protocols appear to be the most important factors to limit or avoid potential negative effects on the periodontium caused by fixed and removable prostheses.

Similarly, Al-Sinaidi and Preethanath [26] assessed the periodontal status of Saudi adult females who had received fixed partial dentures, and the effects of sub- and supra-gingivally placed crown margins were also assessed. They found that the abutment teeth scored significantly higher regarding the plaque and gingival indices and a greater probing pocket depth compared to non-abutment teeth (*p*-value < 0.05). In addition, the abutment teeth attained the highest values in clinical parameters in subjects who were 46 years old or older and in those who had their functioning fixed partial dentures for more than 5 years. The teeth with supra-gingivally placed crown margins had significantly higher mean values of plaque index, gingival index, and probing pocket depth than teeth with sub-gingival crown margins (*p*-value < 0.05). They concluded that the abutment teeth in fixed partial dentures are more prone to periodontal inflammation than the non-abutment teeth. Additionally, the individual’s age, duration of insertion of fixed partial dentures, and location of the crown margins affect the periodontal health of the abutments.

Our study is also supported by Abduo and Lyons [7], who mentioned that although periodontal factors do not usually have a direct effect on the survival of fixed prostheses, a harmony between the prosthesis and the periodontium is critical, otherwise the aesthetics and the longevity of the prosthesis and the periodontium will be compromised. The location of the preparation margin and the contour and the emergence profile of the prosthesis will influence the response of the gingival tissues to the prosthesis. Pontic design and cleansibility also contribute to the response of the gingival tissues as well as to the clinical and aesthetic outcome. Even an optimal pontic design will not prevent the inflammation of the mucosa adjacent to the pontic if pontic hygiene is not maintained by the removal of the plaque. A case selection and the patients’ ability to carry out adequate oral hygiene are therefore essential for the longevity of the prosthesis, and regular reviews provide an opportunity for early detection and treatment of failures.

The fixed prostheses may result in inflammation, and when the inflammation becomes chronic, the adaptive immune response is activated, with the involvement of the cellular and non-cellular mechanisms of acquired immunity. Immune mechanisms play further roles in the resolution of the inflammation and in the healing process, including the repair and the regeneration of lost or damaged tissues. Thus, innate (inflammatory) immunity and acquired immunity must be coordinated to return the injured tissue to homeostasis [27].

The initial lesion occurs in the response of resident leukocytes and endothelial cells to the bacterial biofilm on prosthesis margins. The metabolic products of bacteria trigger junctional epithelium cells to produce cytokines and stimulate neutrons to produce neuropeptides, which cause the vasodilatation of local blood vessels. The early lesion follows, with increased numbers of neutrophils in the connective tissue and the appearance of macrophages, lymphocytes, plasma cells, and mast cells. When the lesion is established, there is a transition from the innate immune response to the acquired immune response. Macrophages, plasma cells, and T and B lymphocytes are dominant, with IgG1 and IgG3 subclasses of B lymphocytes also present. Blood flow is impaired, and the collagenolytic activity is increased. There is also increased collagen production by fibroblasts. Clinically, this stage is a moderate to severe gingivitis with gingival bleeding and color and contour changes. The final stage is the transition to periodontitis: the advanced lesion. Irreversible attachment loss and bone loss are histologically and clinically observed. The inflammatory lesion extends deeper, affecting the alveolar bone and resulting in a periodontal pocket [27,28].

In the present study, the patients with fixed dental prostheses fabricated by CAD/CAM technique had better periodontal outcome compared to the conventionally fabricated dental prostheses. These prostheses perform in a highly complexed oral environment and subject to uncontrollable factors such as masticatory stresses, diversity pH and temperature changes. Therefore, the performance of prosthesis may be influenced by materials, manufacturing techniques and operator or patients related factors. The CAD/CAM fabricated prostheses are biocompatible and perform with an acceptable periodontal response at the tissues and cellular level [29,30,31]. Pabst et al. [30] investigated the influence of four different CAD/CAM all-ceramic materials (e.max CAD LT, e.max CAD HT, Empress CAD and Mark II) for cells viability, migration ability and secretion of phosphotransferase enzyme adenylate kinase (ADK) by human gingival fibroblasts (HGF) and oral keratinocytes (HOK). HGF and HOK were cultured on disc-shaped CAD/CAM all-ceramic materials and on discs made of tissue culture polystyrene surface (TCPS) serving as control. It was reported that there was no significant differences in cell viability and migration ability of HGF and HOK on CAD/CAM all-ceramic materials [30]. These results suggested a high biocompatibility of all the CAD/CAM prosthesis and had no significance influence on ADK secretion by HGF and HOK hence maintaining their energy hemostasis. In contrast, the conventional casting alloys for fabricating metal-ceramic restoration are composed of base metals (mainly cobalt, chromium and nickel) and associated with a number of biocompatibility issues affecting the clinical outcome [31,32,33]. Presence of electrolytes (such as sodium, chloride) may result in release of corrosion products (cations) affecting tissues in contact with the alloy as well as systemic effects such as immune response [34]. The extent of corrosion and associated effects may vary depending on the composition of casting alloy. For example, nickel free alloys are less susceptible compared to the nickel containing alloys [32]. However, there are no evidence suggesting any mutagenic or carcinogenic potential of the dental casting alloys.

In contrast, dental zirconia is a ceramic material that is highly biocompatible and stable while performing under the harsh conditions of the oral cavity [35,36]. Shang et al. [37] studied the effect of CAD/CAM zirconia all-ceramic crown restoration on the state of periodontal health. They compared the CAD/CAM zirconia all-ceramic crowns vs. the control (Ni-Cr alloy porcelain-fused-to metal restorations). The volume of gingival crevicular fluid and the levels of interleukin-6 and tumor necrosis factor-α in the 2 groups were examined at the pre-restoration and post-restoration stages. At 12 months after restoration, in the control group, the volume of gingival crevicular fluid, the levels of interleukin-6 and tumor necrosis factor-α, sulcus bleeding index, probing depth, and plaque index were all increased (*p* > 0.05). These findings suggested that the CAD/CAM zirconia all-ceramic crown restoration is more favorable to the health of periodontal tissues.

Cytomorphometric analysis is a cytological study that may enable the selection of the most appropriate prostheses for patients with existing periodontal pathologies [38,39]. Recently, Heboyan et al. [39] assessed the inflammation dynamics using a cytomorphometric analysis of the periodontium before and after the use of fixed dental prostheses and reported no significant changes in the parameters among patients with a healthy periodontium, before and after prosthetic treatment and regardless of the prosthesis type used. In all study groups, there was a statistically significant increase (*p* < 0.05) in the oral epithelial cell counts and a statistically significant decrease (*p* < 0.05) in the PMN count following the insertion of the fixed prostheses.

The most common complaints among patients after the rehabilitation with conventionally fabricated metal-ceramic prostheses was the presence of a dark rim around the crown margin, a discoloration of the marginal gingiva and papilla, and gingivitis, which was confirmed by clinical examination. However, these signs were not observed when metal-ceramic prosthesis were fabricated by the CAD/CAM technique, which is probably due to a better marginal fit of the metal frame [9]. In the present study, all the symptoms were absent in patients provided with zirconia-based prostheses, since the zirconia does not irritate the soft tissues and promotes the active protection of soft periodontal tissues. In addition, clinical improvement was also observed at the finish line of zirconia-based prostheses. All these effects are attributed to less bacterial adhesion to zirconia compared to metal [40]. The results obtained on studying the dynamics of the amount of gingival crevicular fluid before and after the restoration of teeth with various prosthetic constructions revealed that the best improvement in the indices is observed while using the CAD/CAM-fabricated zirconia-based and cobalt-chromium metal-ceramic prosthesis [41].

Prior to prosthetic treatment, no significant differences in CPI scores were found among the study groups (*p* > 0.1). Long-term results regarding the influence of the use of the prostheses based on the used materials and technologies on periodontal tissues were traced in patients 12 months after the rehabilitation. A significant difference was noted in the following indicators: number of healthy sextants, which was greater in the Group-III patients with periodontitis than in Group-I and Group-II patients (*p* < 0.01 and *p* < 0.05, respectively). Similarly, the number of sextants with a periodontal pocket of 4–5 mm was lower in Group-III patients with periodontitis compared to Group-I (*p* < 0.05). Statistical differences with respect to the presence of signs such as bleeding, dental plaque, periodontal pockets (6 mm or more), and excluded sextants were not observed among all the studied groups, which was also justified by the clinical results. 

Basynet et al. [42] studied the oral hygiene and gingival condition in patients after the placement of a fixed dental prosthesis for a period of 6 months. It was also analyzed how factors like the type of fixed dental prosthesis (single crown, fixed partial denture) and material (metal, porcelain fused with metal) are statistically associated with oral hygiene and gingival health. Teeth and gingiva were examined using the Plaque and Gingiva Index by Silness and Löe. The examinations were conducted after 14 days and 6 months after the placement of a fixed dental prosthesis along with the oral hygiene instructions. No significant difference in plaque index was found among patients with a single crown, whereas the fixed partial denture showed statistical significance. No significant differences were found for the type of material. The statistical analysis showed similar results for the gingival index. It was concluded that the single crown had no significant difference on the Plaque index and Gingival index of the patient after 14 days and 6 months, whereas the fixed partial denture showed a significant difference. Neither the metal crown nor the crown made of porcelain fused with metal revealed statistically significant differences on the Plaque index and Gingival index.

Finally, the present study reported that the gingival biotype plays an important role in the outcome of prosthetic rehabilitation, which agrees with the previous studies [43,44,45]. The knowledge of the nature of tissue biotypes contributes to minimizing tissue resorption and providing a better outcome in tooth preparation and gum retraction. Inappropriate tooth preparation and violation of the biological width can lead to a change in gingival thickness, resulting in a thin biotype over time. It was found that the gingival biotype may undergo a transformation in response to prosthetic rehabilitation from a thick to a thin gingival biotype over time [46]. The thin gingival biotype has a higher susceptibility toward gingival recession, and supragingival margins should be placed wherever possible.

The rough margin of the fixed prostheses can lead to the attachment of bacteria. These can compromise the oral hygiene and lead to gingivitis and periodontitis. Popa et al. [46] studied the zirconia surface modifications occurring after laser irradiation and studied their roughness. Group 1 was irradiated using neodymium: yttrium-aluminum-garnet (Nd:YAG) dental laser, and Group 2 was irradiated using erbium: yttrium-aluminum-garnet (Er:YAG) dental laser. They found that there were significant differences between Group 1 and Group 2 after irradiations. The Nd:YAG laser irradiation produced significantly higher alterations in the surface roughness of zirconia than Er:YAG.

There are a few limitations in the present study: mainly the smaller sample size, as recruiting more patients was not feasible due to the time factor. The small sample size may have negatively impacted on the statistical significance of differences. In this study, we did not evaluate the correlation between the stages of periodontitis and various fixed prostheses factors, such as margin placement and various types of pontic design which can affect the results. This research can be extended in the future to investigate a larger sample size, a prolonged follow up, the correlation of the stages of periodontitis, and various prosthetic factors (occlusal status, margin design, and pontic design). Our study suggests that the prosthetic treatment of periodontal diseases showed better outcomes when using dental prostheses fabricated by the CAD/CAM technique (Group-II and Group-III) compared to the conventionally fabricated fixed dental prosthesis.

## 5. Conclusions

Patients with fixed dental prostheses fabricated by the CAD/CAM technique had a better periodontal outcome compared to that of the conventionally fabricated dental prostheses. Among CAD/CAM restorations, zirconia-based ceramic constructions demonstrated even better results in terms of periodontal health, reduced inflammation, and oral hygiene maintenance. Moreover, the individual gingival biotype should be considered when planning the prosthetic treatment. The gingival biotype and the depth of the gingival sulcus is to be additionally measured to ensure a proper marginal fit of crowns, to prevent trauma to the periodontal tissues, and to avoid conditions favorable for the colonization of microorganisms.

## Figures and Tables

**Table 1:** Study groups and types of the prostheses used.

Group	Description	Clinical Presentation
Group-I (n = 35)	Received a conventionally fabricated cobalt-chrome (Co-Cr) ceramic prosthesis; conventional wax up copings were converted to metal copings by the lost wax technique followed by porcelain layering.	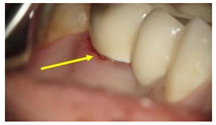
Group-II (n = 30)	Received a Co-Cr ceramic prosthesis fabricated by a computer-aided design and computer aided manufacturing (CAD/CAM) using Sintron technology. Copings were milled from a soft pre-sintered Co-Cr alloy by CAD/CAM, sintered in a special oven and followed by porcelain layering.	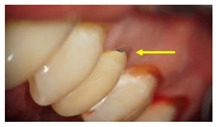
Group-III (n = 30)	Received zirconia-based prostheses fabricated by the CAD/CAM technique. Zirconia was milled using the CAD/CAM system from pre-sintered zirconia blocks, sintered, and followed by porcelain layering.	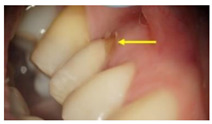

Arrows show the crown margins and gingival tissues interface.

**Table 2:** Modified Approximal Plaque Index (MAPI) index interpretation [19].

% Dental Plaque	Classification
<30	Good oral hygiene
30–60	Moderately poor oral hygiene
60–100	Poor oral hygiene

MAPI scoring codes: 0 (no plaque), 1 (isolated islands or a thin plaque line), 2 (a solid thin plaque area), 3 (plaque of the total interproximal area).

**Table 3:** MAPI data in all treatment groups before and after prosthetic treatment.

Groups	Condition	Before Prosthetic Treatment	After Prosthetic Treatment (12 Months)
Group-I	Healthy	75.17% ± 7.9 (*p* < 0.05)	70.3% ± 8.4 (*p* < 0.05)
	Gingivitis	86.67% ± 4.8 (*p* < 0.01)	76.4% ± 17.4 (*p* < 0.01)
	Periodontitis	94.75% ± 5.3 (*p* < 0.01)	87.9% ± 15.4 (*p* < 0.01)
Group-II	Healthy	71.95% ± 38.2 (*p* < 0.01)	51.8% ± 30.9 (*p* < 0.01)
	Gingivitis	76.4% ± 28.8 (*p* < 0.01)	44.8% ± 26.7 (*p* < 0.01)
	Periodontitis	96.5% ± 8.6 (*p* < 0.01)	80.6% ± 17.97 (*p* < 0.01)
Group-III	Healthy	71.8% ± 39.8(*p* < 0.001)	41.3% ± 28.2 (*p* < 0.001)
	Gingivitis	84.9% ± 32.6 (*p* < 0.003)	40.7% ± 21.4 (*p* < 0.05)
	Periodontitis	93.3% ± 13.9 (*p* < 0.003)	62.5% ± 21.4 (*p* < 0.01)

**Table 4:** Prevalence of gingival biotype in the observation groups.

Gingival Biotype	Group-I			Group-II			Group-III		
	WG	WP	Healthy	WG	WP	Healthy	WG	WP	Healthy
Thin	100	59.3	33.3	88.9	62.5	75	71.4	66.7	76.9
Thick	-	40.7	66.7	11.1	37.5	25	28.6	33.3	23.1

WG = with gingivitis; WP = with periodontitis.

## Data Availability

Not available.

## Sample Availability

Not available.
